# Surface-grafted cellulose particles with poly(butylene succinate) and poly(butylene adipate-*co*-terephthalate) for sustainable composites

**DOI:** 10.1039/d5ra04324g

**Published:** 2025-07-21

**Authors:** Yuuki Takatsuna, Erik Reimhult, Ronald Zirbs

**Affiliations:** a Institute of Colloid and Biointerface Science (ICBS), Department of Natural Sciences and Sustainable Resources, BOKU University Muthgasse 11 1190 Vienna Austria ronald.zirbs@boku.ac.at

## Abstract

The growing need to reduce plastic waste has prompted the development of bio-based and biodegradable materials. Cellulose is attracting increasing attention as a sustainable filler candidate due to its renewability, abundance, and favorable mechanical properties. Its application in polylactic acid (PLA)-based composites has been extensively studied and has demonstrated improvements in mechanical strength, barrier properties, and processability. However, the use of nanocellulose in other biodegradable polymers such as poly(butylene succinate) (PBS) and poly(butylene adipate-*co*-terephthalate) (PBAT) remains limited, despite their industrial significance. In this study, we developed matrix-adapted cellulose nanoparticles by grafting PBS or PBAT onto cellulose regenerated from aqueous sodium hydroxide solution. Grafting was carried out *via* melt polycondensation, resulting in nano-sized particles with average sizes of approximately 100 nm for PBS and 175 nm for PBAT. These surface-modified particles exhibited improved thermal stability and high polymer content, reaching 25 wt% for PBS and 50 wt% for PBAT, indicating successful grafting, which is expected to facilitate compatibility with the target biodegradable matrices. This work provides a new approach for the rational design of biodegradable nanocomposites beyond PLA and contributes to the development of sustainable high-performance materials.

## Abstract 2

PBS/PBAT-grafted cellulose nanoparticles (∼100–175 nm) increase thermal stability and compatibility and enable sustainable, high-performance biodegradable nanocomposites beyond PLA and promote the reduction of plastic waste.

## Graphical abstract

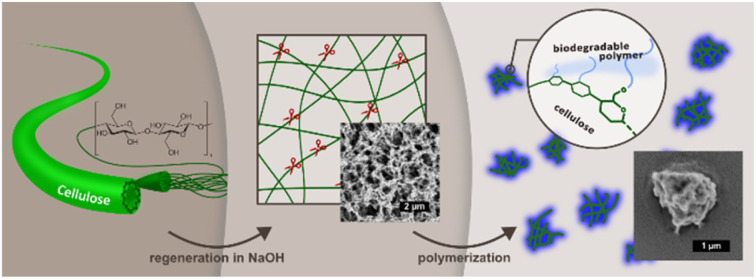

## Introduction

Bio-based and biodegradable nanomaterials have recently gained attention amid growing awareness of environmental issues.^1,2^ These materials are employed as filler material in a composite material with biodegradable polymers, which have the potential to reduce environmental pollution caused by disposable products such as food packaging and agricultural mulch sheets.

In this context, cellulose is one of the most promising filler materials. Cellulose is the most abundant organic compound on Earth. Its physical properties, including high strength, elasticity, and thermal stability, have attracted considerable attention from researchers. While wood and plants harvested for the purpose remain the traditional sources of cellulose, recent studies have investigated the potential of utilizing byproducts of plant-based products that are currently discarded, including bacterial cellulose and fruit peels.^3,4^ Exploiting waste cellulose as a resource doubly contributes to enhanced sustainability.

Due to their superior modification efficiency, nanoscale cellulose materials have attracted considerable attention as promising polymer fillers.^5^ These include cellulose nanocrystals (CNC), cellulose nanofibers (CNF), and bacterial cellulose (BC), which are produced *via* acid hydrolysis, mechanical and/or chemical defibrillation, and bacterial biosynthesis, respectively.^6^ However, the inherently high hydrophilicity of cellulose leads to poor dispersibility in hydrophobic polymer matrices, making it difficult to achieve uniform distribution. Therefore, surface modification is essential to improve compatibility and performance in such systems.^7^

Many studies on cellulose fillers have been reported, and composite materials have been developed to overcome these problems. For instance, He *et al.* showed that the surface modification of CNC improved its dispersion in poly(butylene succinate) (PBS), resulting in an increased tensile modulus of the composite.^8^ Niu *et al.* demonstrated that a composite material comprising poly(lactic acid) (PLA) and CNC grafted with aliphatic chains exhibited a six-fold increase in elongation at break.^9^ However, many of these modification strategies involve complex procedures that may hinder scalability for industrial applications. Furthermore, the incorporation of surface-modified nanoparticles has also been reported to improve the thermal stability of composite materials.^10–13^

In recent years, several methods have been reported to synthesize cellulose nanoparticles from cellulose solutions.^14,15^ We recently demonstrated that composite materials of PLA and nanocellulose particles surface-modified with oligo-l-lactic acid (OLLA) have improved mechanical and barrier performance.^16^ By covalently grafting a shell with physicochemical properties matching the matrix polymer, the affinity between the filler material and polymer matrix will be improved, reducing aggregation that compromises the properties of the composite material.^7^

While extensive efforts have focused on designing nanocellulose fillers optimized for PLA, little attention has been paid to developing analogous systems for other biodegradable polymers. Nevertheless, matrix polymers differ widely in their surface energy, hydrogen-bonding ability, and crystallinity. As such, the surface of cellulose fillers should be rationally engineered to match the specific interactions of the intended matrix polymer. There is a growing need to design matrix-optimized nanofillers to realize the full potential of biodegradable polymer composites.

Poly(butylene succinate) (PBS) and poly(butylene adipate-*co*-terephthalate) (PBAT) are promising biodegradable polymers that are currently being produced on an industrial scale.^17,18^ PBS is synthesized *via* polycondensation of 1,4-butanediol and succinic acid. It exhibits excellent processability and comparable tensile strength to polypropylene and polyethylene.^19^ PBAT is synthesized *via* polycondensation of 1,4-butanediol, adipic acid, and terephthalic acid. Due to its high elongation at break and flexibility, it is a good candidate for applications in packaging materials, as well as hygiene and biomedical products.^18,20^ Despite the practical importance of PBS and PBAT, the development of nanocellulose fillers optimized for these matrices has received limited attention.

**Fig. 1:**
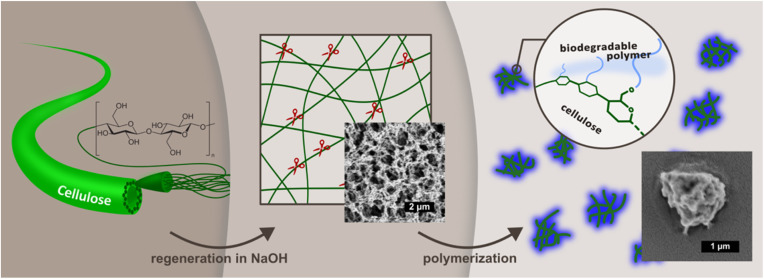
Schematic overview of the synthesis process of surface-modified cellulose particles.

To address this gap, we propose a tailored surface grafting strategy to design matrix-compatible cellulose particles by grafting the respective polyesters onto regenerated cellulose cores (Fig. 1). This approach aims to enhance polymer–filler interactions by introducing chemically matched shells, thereby improving dispersibility and eliminating the need for compatibilizers or excessive mixing energy. Polymer grafting was achieved through melt copolycondensation of the corresponding monomers *via* the grafting-from method, which provides a better grafting density than the grafting-to method.^21^ Moreover, the method established in this study has the potential to be applied as a masterbatch synthesis method, offering a promising avenue for further research and development. The dilution of the masterbatch, consisting of a high load of fillers in a polymer matrix, is a promising method to create a composite material. This method has been demonstrated to enhance the mechanical properties of the resulting composite material in comparison to the conventional solvent casting method. Furthermore, the quantity of solvent used throughout the entire process can be significantly reduced due to the omission of the filler purification process.^22^

The synthesis process established in this study addresses several key chemical and structural transformations: purification, nanoparticulation, and surface activation of cellulose; hydrophobization through polymer grafting. It demonstrates an efficient and industrially scalable synthesis of cellulose nanomaterials from various cellulose sources.

## Experimental

### Materials

1,4-Butanediol (BDO), succinic anhydride (SA), adipic acid (AA), terephthalic acid (TPA), titanium(iv) butoxide (TBT) and sodium hydroxide used in this study were purchased from Sigma-Aldrich and used without further purification. Microcrystalline cellulose (particle size: 10–100 μm) was purchased from Sigma-Aldrich. Bacterial cellulose was produced as a by-product of fermentation using symbiotic culture of bacteria and yeast (SCOBY) during kombucha production.

### Synthesis

#### SCOBY cellulose production and purification

100 mL of fully fermented kombucha tea (sucrose content < 5%) and approximately 10v% of previously produced SCOBY were mixed with 10 g L^−1^ of agricultural waste obtained after boiling of leaves and mixed biowaste for 10 min and adding 100 g L^−1^ of sucrose. The fermentation was carried out for 15 days at room temperature, resulting in SCOBY production with a high content of bacterial cellulose (BC). 1000 g of SCOBY was blended for 1 min using a blender (Nutri-Blender MAX Hochleistungsmixer 2000 W, Munich, Germany) and then centrifuged. The blended BC was re-dispersed in deionized water and centrifuged again. The remaining microorganisms and soluble polysaccharides were removed by stirring in a 0.1 M NaOH aqueous solution at 80 °C for 20 min. The purified white BC was then centrifuged three times, washed to neutral pH, and stored as a 3% dispersion in water (148.5 g in wet mass) in a 2 L beaker.

#### Pretreatment of cellulose with NaOH

The regeneration procedure of cellulose in this study was conducted according to the procedure reported by Isogai *et al.* with minor changes.^23^ A cellulose suspension (26.9 mL per 1 g of cellulose) was homogenized at 25 000 rpm using a homogenizer (IKA T18 basic ULTRA TURRAX, Staufen, Germany). NaOH (2.5 g per 1 g of cellulose) was dissolved in the dispersion, which was then frozen at −24 °C for 48 h. The tight solid mass was transferred from the freezer to room temperature to thaw, and water (20.6 mL per 1 g of cellulose) was added. The resulting cellulose solution in aqueous NaOH was produced by mechanical homogenization of the thawing mixture. The partial disruption of the hydrogen bonds between the nanofibrils resulted in increased accessibility of the OH groups. Cellulose was precipitated by addition of EtOH and neutralized with hydrochloric acid, then washed with water and centrifuged. This process was repeated three times to remove remaining salt. The NaOH-treated cellulose gel was subjected to further grafting reactions. The designation of NaOH-treated microcrystalline cellulose (MCC) as TMCC and NaOH-treated BC as TBC will be employed.

#### Synthesis of PBS modified TMCC (TMCC–PBS)

The PBS modification followed a PBS synthesis method described in previous studies with minor changes.^24^ NaOH-pretreated cellulose gel, prepared from 24 g of MCC, and 100 g of SA (1 eq.) were dispersed in 112 g of BDO (1.2 eq.). The mixture was mechanically stirred at 135 °C and 500 mbar for 3 h to eliminate EtOH and water residue. Then, the temperature was increased to 150 °C and stirred for 2 h. 212 μL of TBT was added and stirred for 3 h at 200 °C. The surface modified cellulose was separated from the crude product by dissolving free PBS in dichloromethane followed by centrifugation. The washing procedure was repeated three times to obtain the final product as light brown powder.

#### Synthesis of PBAT modified TMCC (TMCC–PBAT)

The synthesis of PBAT was conducted in accordance with previous studies on the synthesis method.^25,26^ NaOH-pretreated MCC gel, prepared from 24 g of MCC, were re-dispersed in 63 g of BDO (0.6 eq.) and transferred into a 1 L three-neck flask, and 87.7 g of AA (0.5 eq.) were added. The mixture was mechanically stirred at 120 °C and 200 mbar for 1 h to remove water and EtOH. The temperature was then increased to 160 °C and stirred for 3 h. Meanwhile, 99.6 g of TPA (0.5 eq.), 63 g of BDO (0.6 eq.) and 119 μL of TBT were stirred in a 1 L beaker at 230 °C for 90 min to make TPA–BDO oligomers until the solution was clear. The TPA–BDO oligomer was crushed and added to the cellulose mixture and stirred for 3 h. An additional 119 μL of TBT were added to the mixture, which was then stirred for a further 8 h at 210 °C. The surface modified cellulose was separated from the crude product by dissolving free PBAT in dichloromethane followed by centrifugation. The washing procedure was repeated three times to obtain the final product as brown powder.

#### Synthesis of PBS modified TBC (TBC–PBS) and PBAT modified TBC (TBC–PBAT)

4 g of purified BC was subjected to NaOH-pretreatment, and the same procedures with the same monomer feeding amount described for modification of TMCC were performed for TBC–PBS and TBC–PBAT, respectively.

#### Synthesis of MCC–PBS

30 g of MCC and 87 g of SA (1 eq.) were dispersed in 80 g of BDO (1.02 eq.), and the mixture was mechanically stirred at 135 °C and atmospheric pressure for 30 min. The pressure was reduced to 100 mbar for a additional 1 h. The temperature was increased to 150 °C and stirred for 2 h. 151 μL of TBT was added, and the mixture was stirred at 210 °C for 3 h. The surface modified cellulose was separated from the crude product by dissolving free PBS in dichloromethane followed by centrifugation. The washing procedure was repeated three times to obtain the final product as light brown powder.

#### Synthesis of MCC–PBAT

34.8 g of TPA (0.5 eq.), 54 g of BDO (1.2 eq.) and 51 μL of TBT were mechanically stirred at 230 °C for 2 h at atmospheric pressure. 30.6 g of AA (0.5 eq.) was added to the mixture, which was stirred for 3 h at 100 mbar. The temperature was reduced to 160 °C and 30 g of MCC were added to the mixture and stirred for 1 h. The mixture was then further stirred for 8 h after addition of 51 μL of TBT. The surface modified cellulose was separated from the crude product by dissolving free PBS in dichloromethane followed by centrifugation. The washing procedure was repeated three times to obtain the final product as brown powder.

### Characterizations

#### Wide-angle X-ray diffraction (WAXD)

WAXD was performed on a Rigaku S-Max 3000 with a MM002 + microfocus source (Cu-Kα radiation with *λ* = 1.504 Å, 45 kV) and a 2D Image Plate detector (Fuji) with 100 μm resolution, which corresponds to a resolution in scattering angle 2*θ* of about 0.047°. The samples were put between polymer film for support and measured in vacuum for 2400 s. WAXD images were acquired, integrated, calibrated and background corrected. Peaks observed in the range of 2*θ* = 10–40° in the resulting scattering curves were analyzed to qualitatively assess the crystalline structure of cellulose.

#### Fourier transform infrared spectroscopy (FTIR)

The molecular structure of filler material was confirmed by FTIR using a FT-IR ATR spectrometer (Vertex 70, Bruker Austria GmbH, Vienna, Austria), 32 scans at the wavelength range of 400–4000 cm^−1^ in transmission mode. Substances were directly mounted on the ATR unit and measured with the pressure stamp.

### Thermogravimetric analysis (TGA)

TGA was performed on a Mettler Toledo TGA/DSC (Mettler Toledo GmbH, Vienna, Austria) under nitrogen atmosphere (80 mL per min nitrogen). The measurement was carried out in a temperature range from 25 to 650 °C with a heating rate of 10 °C min^−1^. The resulting DTG diagrams were analyzed using Origin (OriginLab, MA, USA) with the peak fitting function to calculate the fraction of cellulose and grafted polymer. The Asym2sig function was used for peak fitting due to the asymmetric shape of the peaks.

#### Dynamic light scattering (DLS)

The samples were dispersed in dichloromethane and sonicated for 15 min, then filtered using a syringe filter (pore size: 0.45 μm). Then DLS measurements were carried out on a Malvern Zetasizer Nano-ZS (Malvern Panalytical Ltd, Malvern, UK). The concentration of particles in dichloromethane was 1 mg mL^−1^, and the measurements were performed at 25 °C and at least three times.

#### Scanning electron microscopy (SEM)

SEM was employed to examine the surface morphology of the modified cellulose. A series of sample dispersions were prepared in very low concentrations in dichloromethane and subsequently dried on silicon wafers, which were gold sputtered with a sputter coater (LEICA EM SCD005) prior to scanning. High-vacuum secondary electron imaging was performed using an Apreo VS SEM (Thermo Scientific, The Netherlands) at an acceleration voltage of 2–5 kV.

## Results and discussion

### Preparation of polymer-grafted cellulose particles

**Fig. 2:**
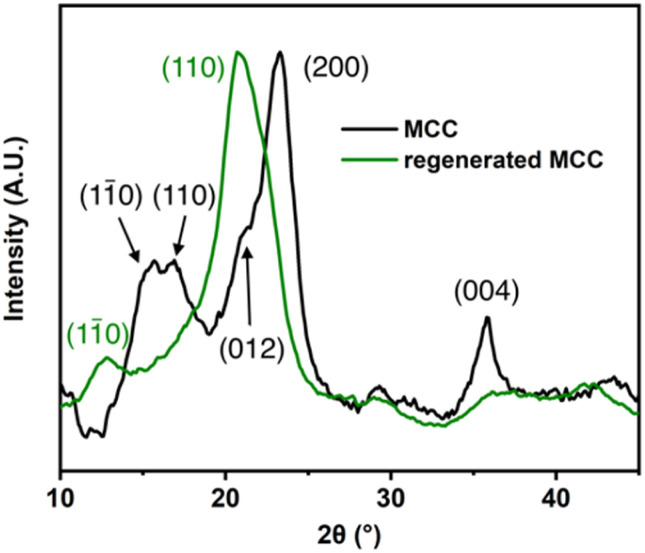
Wide-angle X-ray diffraction (WAXD) patterns of microcrystalline cellulose (MCC) and regenerated MCC after NaOH/ethanol treatment.

Polymer grafting onto regenerated cellulose was achieved *via* melt polycondensation using PBS or PBAT monomers. It is well known that regeneration of cellulose alters its crystalline structure, which can be confirmed by WAXD. As shown in the WAXD diffraction pattern in Fig. 2, MCC exhibits peak maxima at 2*θ* angles of 16.1°, 17.8°, 21.0°, 22.8°, and 35.6°, which are characteristic of cellulose I and correspond to the (1̄10), (110), (012), (200), and (004) planes, respectively.^27^ In contrast, the regenerated MCC shows peaks at 13.2° and 21.0°, which are characteristic of cellulose II and correspond to the (1̄10) and (110) planes, respectively.^27^ The regenerated cellulose exhibits a porous structure, with a larger surface area compared to MCC and a greater number of accessible hydroxyl groups. The surface of this porous cellulose is then modified with PBS and PBAT. It is believed that nanoscale particles are formed from the network structure of the porous body due to shear stress generated during stirring in the modification process, as well as partial hydrolysis caused by heat and acid.

Table 1 shows the characteristics of the cellulose particles synthesized in this study and the cellulose used as the raw material. As described below, FTIR analysis confirmed that the synthesized particles were modified with PBS and PBAT, respectively. TGA analysis revealed that the weight fraction of the polymer in the PBAT-modified samples was approximately 50%, while in the PBS-modified samples, it was approximately 25%. The ratio of the initial cellulose amount to monomer feeding amount for TBC samples was about five times higher than that for TMCC samples. However, the weight fraction of grafted polymer was found to be nearly equal, indicating that the amount of monomers was saturated against the cellulose amount at least at a cellulose/BDO ratio of 20% for the synthesis. As long as the mixing process is feasible, it may be possible to reduce the monomer feeding amount further, which would allow for the synthesis of masterbatches with higher cellulose filler concentrations. This hypothesis will be tested in future since our setup did not allow further monomer reduction.

**Table 1:** A list of the properties of raw cellulose and synthesized cellulose particles. Weight fraction of initial cellulose against BDO feed (C/B), reaction time of second stage (*R*_*t*_), weight fraction of polymer (*W*_g_), hydrodynamic diameter (*D*_h_) and 5% weight loss temperature (*T*_5%_)^a^

Sample	Cellulose	Pre-treatment	Polymer	C/B [%]	*R*_*t*_ [h]	Yield^b^ [%]	*W*_g_ [%]	*D*_h_ [nm]	PDI	*T*_5%_ [°C]
MCC	MCC	—	—	—	—	—	—	10–100 μm^d^	—	305.5
MCC–PBS	MCC	—	PBS	38	3	68	—c	—e	—	297.5
MCC–PBAT	MCC	—	PBAT	38	5	63	—c	—e	—	309.6
TMCC	MCC	✗	—	—	—	—	—	—e	—	289.5
TMCC–PBS	MCC	✗	PBS	21	3	66	24.9	99.2 ± 25.3	0.187	298.6
TMCC–PBAT	MCC	✗	PBAT	19	8	62	51.3	175.3 ± 47.3	0.227	310.5
BC	BC	—	—	—	—	—	—	—e	—	298.8
TBC	BC	✗	—	—	—	—	—	—e	—	281.4
TBC–PBS	BC	✗	PBS	4	3	72	25.8	105.0 ± 18.3	0.282	298.4
TBC–PBAT	BC	✗	PBAT	3	8	68	57.4	175.4 ± 65.1	0.205	292.8

a All samples were synthesized with a catalyst concentration of 5 ppm.

b Yield against the initial amount of cellulose.

c Not detectable by DTG peak analysis.

d Notation by producer.

e Measurement not possible due to the sedimentation.

The reaction employed to modify the cellulose surface is an application of a common polyester synthesis reaction.^19^ Consequently, it may be feasible to modify the cellulose surface with other polyesters by modifying the monomers, either by changing them or by combining them. For instance, polymer blends of PLA and poly(butylene succinate-*co*-terephthalate) (PBST) have been reported to exhibit high barrier performance.^28^ It is believed that cellulose particles modified with PBST can be synthesized by using succinic acid and terephthalic acid as monomers for cellulose modification.

### FTIR analysis

**Fig. 3:**
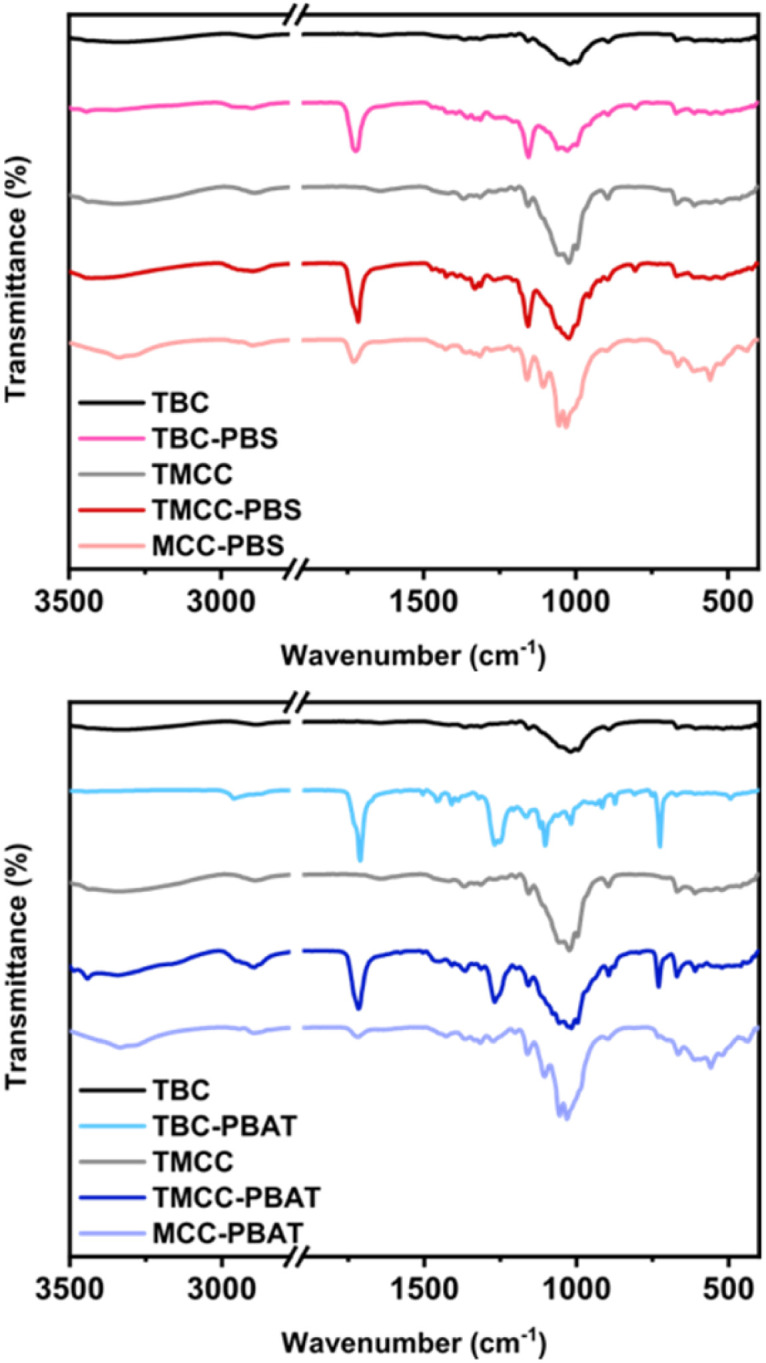
FTIR spectra of PBS-modified cellulose particles (left) and PBAT-modified cellulose particles (right).

The FTIR spectra of regenerated MCC, BC, and modified cellulose samples after purification are shown in Fig. 3. The peaks observed in the FTIR spectra represent the chemical structure of the cellulose particles since free PBS and PBAT, which are by-products of the melt copolycondensation, are removed during the purification process. In comparison to the spectra of regenerated MCC and BC prior to the synthesis reaction, a new peak around 1750 cm^−1^ is observed in the spectra of the post-grafting samples. This peak is assigned to the carbonyl group, which is the fundamental structural unit of PBS and PBAT.

In the spectra of the PBAT grafted sample, the aromatic COO stretching peak is observed at 1200 cm^−1^. Additionally, the in-plane and out-of-plane bending modes of C–H in the benzene ring were observed at 1020, 874, and 730 cm. This demonstrates that the cellulose nanoparticles were modified with PBS or PBAT.

Comparing the two samples with and without NaOH pretreatment after normalization by the peak that characterizes cellulose, the sample with pretreatment shows a stronger carbonyl peak. This observation suggests that the NaOH pretreatment significantly increased the surface area of the cellulose, enhancing accessibility to OH groups and leading to a higher grafting amount.

#### Thermogravimetric analysis

**Fig. 4:**
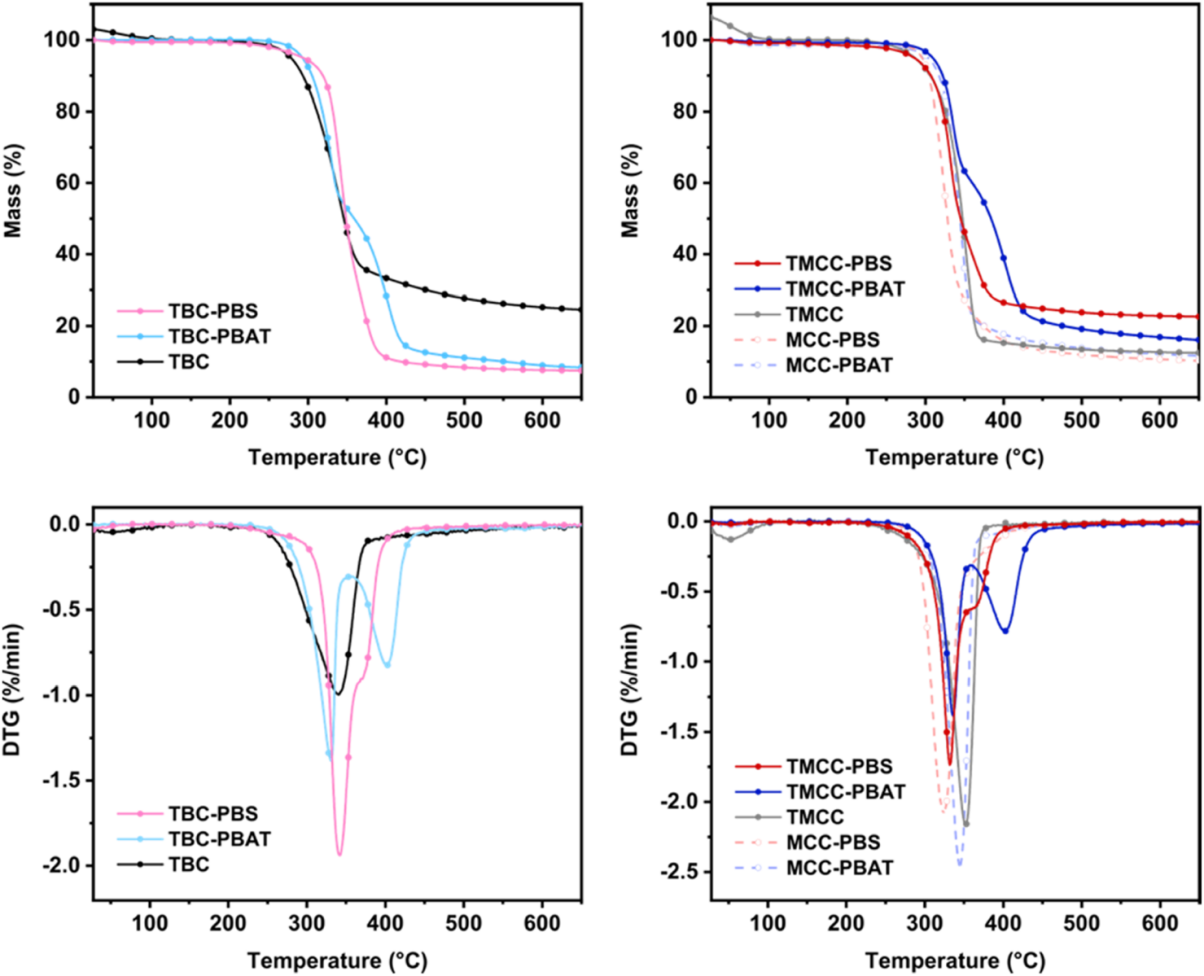
Diagrams of thermogravimetry data and its derivative for bacterial cellulose-based particles (left) and microcrystalline cellulose-based particles (right).

Thermogravimetric analysis was conducted to assess the thermal stability of the product and to estimate the quantity of grafted polymer.^29,30^ It is crucial to assess the thermal stability of the synthesized cellulose particles to consider their applicability in the production of composite materials with polymers. The processing temperatures of many polymeric materials exceed 200 °C. The temperature, *T*_5%_, at which a 5% weight loss occurred was used to indicate the thermal stability of the particles (Fig. 4). Both MCC- and BC-based particles with polymer grafting exhibited an increase in *T*_5%_ by approximately 10 to 20 °C compared to the samples without grafting.

The amount of grafted polymer was determined by integrating the DTG peaks. The peaks observed in the range of 200 to 500 °C, which are attributed to the degradation of cellulose and grafted polymers, were separated using the peak analyzer function, and the weight ratios were derived from the peak area ratios. The difference observed in the residue after combustion between BC and MCC is consistent with findings in other studies and is thought to be due to the high crystallinity of bacterial cellulose and its function as a flame retardant.^31,32^ The results are presented in Table 1. The samples without NaOH pretreatment exhibited no quantifiable peak of grafted polymer. This agrees with the FTIR analysis showing that the grafting is lower in the untreated, more crystalline samples. This result indicates that, in the absence of NaOH pretreatment, the polymer was grafted onto the surface of the cellulose particles. The NaOH pretreatment increased the number of accessible hydroxyl groups, resulting in more polymers being grafted.

### Particle size and morphological analysis

**Fig. 5:**
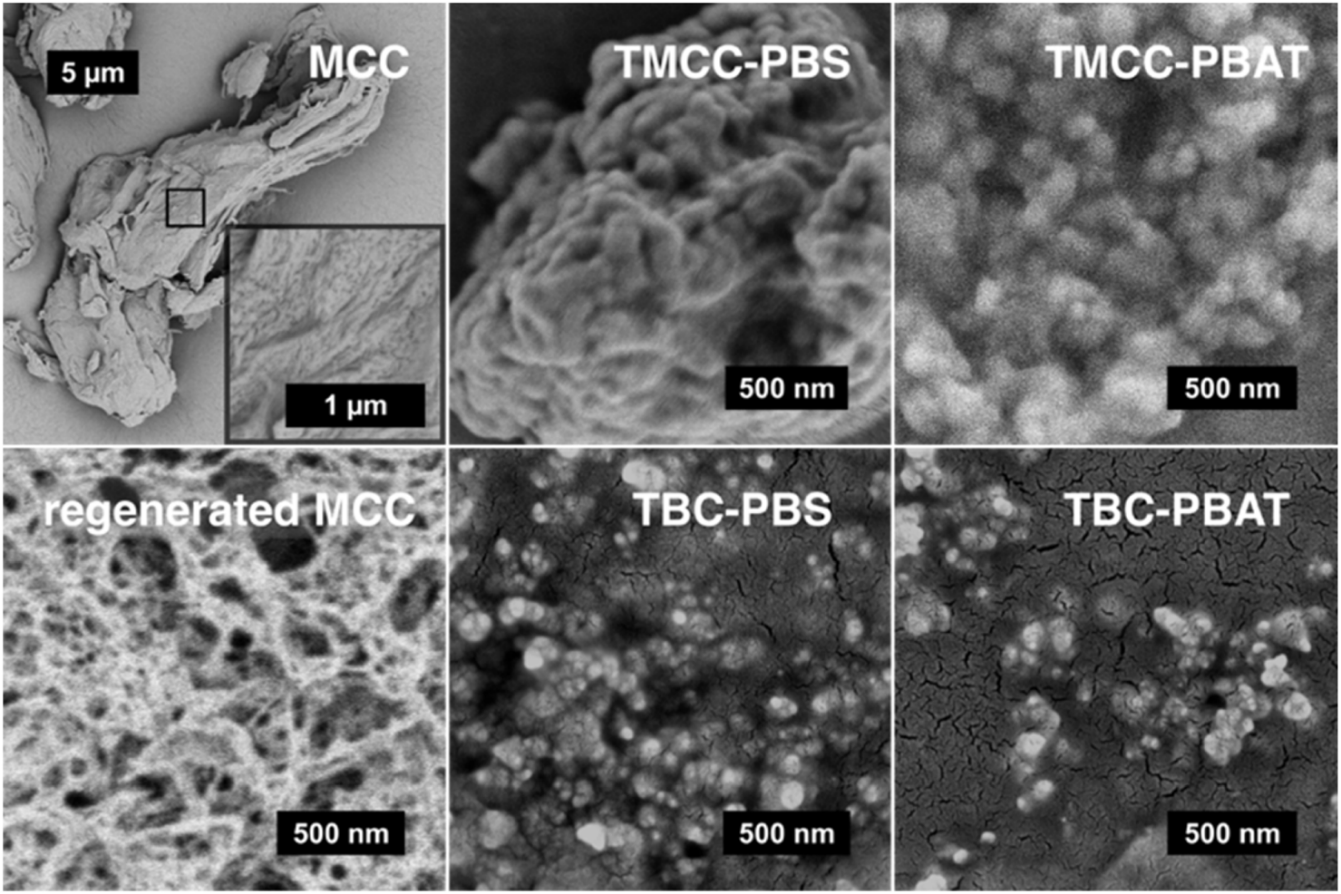
SEM images of cellulose samples at different modification stages. Structural transition of MCC from irregular, micron-sized particles to a porous, network-like morphology after regeneration is shown on the left. The center and right images display agglomerates of nanosized cellulose particles surface-modified with PBS and PBAT, respectively.

**Fig. 6:**
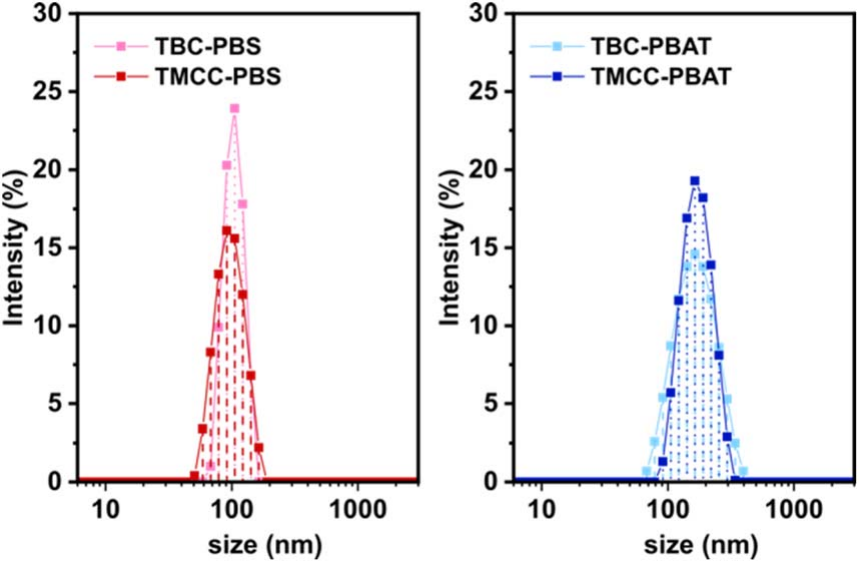
Intensity-weighted size distribution of PBS-modified cellulose in dichloromethane (left) and PBAT-modified cellulose in dichloromethane (right).

The morphological characterization of the cellulose particles was conducted using SEM, and the results are shown in Fig. 5. MCC, with a size of several tens of μm, is dissolved in NaOH aqueous solution and regenerated by adding ethanol, leading to a structural transformation into porous regenerated cellulose, as shown in the SEM images. The observed reduction in size is attributed to mechanical shear during stirring and partial hydrolysis under heat and acidic conditions, which likely contribute to the breakdown of the porous regenerated structure into nanosized particles. The analysis revealed the presence of agglomerates of cellulose particles in all modified samples with NaOH pretreatment. As shown in Fig. 6, the hydrodynamic size distribution of cellulose particles in dichloromethane was determined *via* DLS, indicates that all samples, with grafted polymers, can exist as submicron-sized particles in solvents capable of dissolving the grafted polymers. Surface-modified cellulose particles with NaOH pretreatment exhibited a narrow monomodal distribution with a PDI < 0.3. The PBS-modified sample had an average particle size of approximately 100 nm, whereas the PBAT-modified cellulose particles showed an average size of approximately 170 nm. These nano-sized particles were not detected in the samples without NaOH pretreatment. This result confirmed the necessity of pretreatment with NaOH for the fabrication of nano-sized cellulose particles. The hydrodynamic size of the PBAT-modified cellulose in dichloromethane is approximately 75 nm larger than the PBS-modified cellulose, corresponding to a five-fold increase in volume. The observed increase in size cannot be explained solely by the difference in polymer content measured in the TGA analysis, even when considering the 1.2-fold difference in density between PBAT and PBS. The higher polymer content does suggest that a larger fraction of the cellulose surface was grafted with PBAT, potentially forming a more solvent-accessible shell. Several factors could have contributed to the larger hydrodynamic size observed for the PBAT-modified cellulose particles, including differences in polymer chain length, potential particle aggregation, and swelling due to solvent uptake. Moreover, cellulose is a naturally hydrophilic material with a high swelling capacity. Espino-Pérez *et al.* propose that surface-functionalized CNC adsorbs organic solvents, causing cellulose to swell.^33^ In this case, the PBAT-modified cellulose may have taken up more solvent molecules, even within the internal structure of cellulose. This could have resulted in particle swelling and the observed larger hydrodynamic size.

## Conclusions

Nano-sized, surface-modified cellulose particles were successfully synthesized from regenerated cellulose using the grafting-from method. Fourier-transform infrared spectroscopy (FTIR) and thermogravimetric analysis (TGA) confirmed the grafting of biodegradable polymers, poly(butylene succinate) (PBS) and poly(butylene adipate-*co*-terephthalate) (PBAT), onto the cellulose particles. Dynamic light scattering (DLS) analysis indicated a narrow monomodal size distribution, with average particle sizes of approximately 100 nm for PBS-modified and 175 nm for PBAT-modified cellulose particles. Scanning electron microscopy (SEM) revealed a spherical morphology of the synthesized particles. TGA results showed a grafted polymer content of about 25% by weight for PBS-modified and 50% for PBAT-modified particles, in addition to indicating the potential use of bacterial cellulose as a flame-retardant component due to its high char residue.

Although the biodegradability of the final composite particles was not evaluated in this work, the use of PBS and PBAT as biodegradable grafting polymers offers a promising foundation for the development of environmentally compatible materials. This work demonstrates a tailored surface grafting strategy to fabricate matrix-compatible cellulose nanomaterials. The demonstrated synthesis approach is not only effective for PBS and PBAT grafting but also holds potential for application to other polyesters, many of which are biodegradable. The NaOH pretreatment method enhances cellulose grafting capacity and simultaneously could serve as an efficient purification step for cellulose, making it applicable for various cellulose materials, including those derived from agricultural waste. Our approach paves the way for the sustainable and large-scale production of high-performance biodegradable polymer composites.

## Author contributions

Yuuki Takatsuna and Ronald Zirbs conceived the ideas. Yuuki Takatsuna conducted material preparation, data collection and evaluation and wrote the first draft of the manuscript. Ronald Zirbs supervised, and Erik Reimhult co-supervised the project.

## Conflicts of interest

The authors have no competing interests to declare that are relevant to the content of this article.

## Data Availability

All data supporting the findings of this study are included within the article. No additional datasets or supplementary information are available.
